# Suppression of the activity of arbuscular mycorrhizal fungi by the soil microbiota

**DOI:** 10.1038/s41396-018-0059-3

**Published:** 2018-01-30

**Authors:** Nanna B Svenningsen, Stephanie J Watts-Williams, Erik J Joner, Fabio Battini, Aikaterini Efthymiou, Carla Cruz-Paredes, Ole Nybroe, Iver Jakobsen

**Affiliations:** 1 Department of Plant and Environmental Sciences, University of Copenhagen, Frederiksberg, Denmark; 2 School of Agriculture, Food and Wine, University of Adelaide, Glen Osmond, SA Australia; 3 Norwegian Institute for Bioeconomy Research, Ås Akershus, Norway; 4 Department of Agriculture, Food and Environment, University of Pisa, Pisa, Italy

## Abstract

Arbuscular mycorrhizal fungi (AMF) colonise roots of most plants; their extra-radical mycelium (ERM) extends into the soil and acquires nutrients for the plant. The ERM coexists with soil microbial communities and it is unresolved whether these communities stimulate or suppress the ERM activity. This work studied the prevalence of suppressed ERM activity and identified main components behind the suppression. ERM activity was determined by quantifying ERM-mediated P uptake from radioisotope-labelled unsterile soil into plants, and compared to soil physicochemical characteristics and soil microbiome composition. ERM activity varied considerably and was greatly suppressed in 4 of 21 soils. Suppression was mitigated by soil pasteurisation and had a dominating biotic component. AMF-suppressive soils had high abundances of Acidobacteria, and other bacterial taxa being putative fungal antagonists. Suppression was also associated with low soil pH, but this effect was likely indirect, as the relative abundance of, e.g., Acidobacteria decreased after liming. Suppression could not be transferred by adding small amounts of suppressive soil to conducive soil, and thus appeared to involve the common action of several taxa. The presence of AMF antagonists resembles the phenomenon of disease-suppressive soils and implies that ecosystem services of AMF will depend strongly on the specific soil microbiome.

## Introduction

Soil microorganisms are fundamental for soil health and provide ecosystem services that are essential for plant production [1, 2]. Societal awareness of agricultural sustainability is continuously growing, making it increasingly important to unravel the biotic and abiotic factors in different soils that support the beneficial components of their microbiome.

Arbuscular mycorrhizal fungi (AMF) are present in most soils and form symbiotic associations with majority of crop plants [3]. The mycorrhizal symbiosis has multiple beneficial impacts on nutrient cycling and plant stress tolerance [4] and represents under-exploited potential to increase global food security [5]. Hence, a well-developed extra-radical mycelium (ERM) that proliferates in the bulk soil beyond the rhizosphere is important for plant P uptake, while the AMF depend on their host plant for carbon nutrition [6, 7]. The large surface area of the ERM provides nutrient-rich niches for colonisation and growth of other soil microorganisms, in particular bacteria [8]. Hence, several members of the Glomeraceae increase the abundance of Firmicutes, Streptomycetes and members of the family Oxalobacteraceae on hyphae, or in soil affected by hyphae [9–11]. The hyphae-associated bacteria may in return affect the AMF as exemplified by mycorrhizal helper bacteria that promote AMF hyphal growth and root colonisation [12, 13]. Yet, even bacteria with suppressive effects have been identified [14, 15]. These observations support the notion that soil harbours both stimulatory and antagonistic bacteria towards AMF. However, we know relatively little about how AMF hyphae and bacteria interact in soil outside the rhizosphere, even though those hyphae represent the major niche where interactions with soil bacteria can take place [10].

Scattered evidence suggests that natural soils can suppress AMF colonisation and plant growth responses ([16]; see also review by Nehl et al. [17]). In one study, the addition of a bacterial soil filtrate decreased the length of the ERM [18]. Furthermore, the contribution of the ERM pathway to plant P uptake was negligible in one out of five unsterile soils, which otherwise produced similar levels of root colonisation [19]. These observations led us to speculate that soils may be either suppressive or conducive to ERM activity. Whereas soils suppressive towards plant pathogenic fungi, so-called ‘disease-suppressive soils’, are well-known [20], it remains unknown whether soil-induced suppression of AMF is common. Disease suppression may be related to the total microbial community in the soil (general suppression), while in other cases, suppression is caused by the specific effects of selected soil microorganisms (specific suppression) [21]. Thus, soil pasteurisation strongly reduces both general and specific disease suppression of soils [22, 23], while transferability of small soil volumes is a key characteristic of specific suppression [21]. Importantly, suppressive effects mediated by components of the soil microbiota might well be confounded by specific characteristics of the soil physicochemical environment such as low temperature [24] or low pH [25, 26].

Microbial community analysis has revealed that suppression of fungal root pathogens may be associated with groups of bacteria or fungi in the rhizosphere microbiome [22, 27, 28]; Actinomycetes and *Lysobacter* strains are examples of *Rhizoctonia*-suppressive bacteria [29]. Interestingly, microbial consortia appear to be more suppressive than individual microbial isolates [20, 30].

The aim of this work was to test the following hypotheses: (1) that field soils exert differential effects on the activity of the ERM of AMF; and (2) that the soil contains microbial populations responsible for suppression of the ERM activity. To address these aims, 21 cultivated soils were screened for their ability to suppress AMF in a model system where P uptake from radioisotope-labelled soil was used as a proxy for activity of ERM. The ERM activity was related to a range of soil physicochemical characteristics and to the composition of the soil microbiome.

## Materials and methods

### Soils, plant and AMF

Plants grew in soil collected from the Risø field site at the Technical University of Denmark (referred to as ‘Risø’ hereafter; Table 1). The soil was γ-irradiated (15 kGy) and mixed 1:1 with quartz sand (*w*/*w*) and with basal nutrients as in ref. [31]. This semi-sterile standard soil was used in all pots except in Expt. 1 where half of the pots contained the corresponding unsterile soil. Soils used in hyphal compartments (HC) of the plant model system (see below) were collected from 21 Scandinavian field sites and had contrasting organic matter content, texture, pH and plant-available P concentrations (Table S1). The soils were air-dried, sieved and stored dry; one soil (‘Risø stored’) had been collected in 1982 and was subsequently stored dry.

Two different AMF were used as inoculum: experiment 1 (see below) used *Funneliformis mosseae* (Nicolson & Gerd.) C. Walker & A. Schüßler (BEG85), formerly known as *Glomus mosseae*, and experiments 2 and 3 used *Rhizophagus irregularis* (Blaszk., Wubet, Renker & Buscot) C. Walker & A. Schüßler (BEG87), f.k.a. *Glomus intraradices*. Both inocula consisted of dry soil, spores and root fragments from *Trifolium subterraneum* pot cultures. Mycorrhizal associations were established with *Medicago truncatula* cv. Jemalong (A17). Plants received supplemental N during the course of growing to suppress nodulation by *Rhizobium*.

### Model system

The model system is modified from ref. [32] (Figure S1). *M. truncatula* plants colonised by AMF served as donors for the production of ERM. The system enables studies of the ability of ERM to colonise, and take up P from, a mesh-enclosed soil patch (the ‘HC’ that was buried in pots with the standard Risø soil supporting plant growth). The HC soil was sieved (<2 mm), mixed 1:1 with quartz sand and labelled with a radioisotope of P. The plant uptake of the radioisotope at harvest was subsequently quantified and used as a proxy for the P uptake activity of the ERM. The HCs were 50 mL plastic cylinders capped at both ends with 25 μm nylon mesh, which allowed for ingrowth of ERM, but not roots.

### Experiment 1: do field soils suppress ERM production and activity?

A dual radioisotope labelling experiment was conducted to test whether ERM activity differs between semi-sterile and unsterile soil. Three pre-germinated *M. truncatula* seeds were sown into each pot, which contained 1.5 kg standard soil mixed with 20 mg P per kg (as KH_2_PO_4_). Half the pots contained semi-sterile soil, while the other half contained unsterile soil. The soil in half of each of the semi-sterile and unsterile pots was mixed thoroughly with 75 g of *F. mosseae* inoculum. Two HCs were placed in each pot; both contained 55 g of standard soil, either semi-sterile or unsterile that had been uniformly mixed with either 5 kBq g^−1^ soil of carrier-free H_3_^33^PO_4_ (for semi-sterile soil) or 5 kBq g^−1^ soil of carrier-free H_3_^32^PO_4_ (for unsterile soil). Each treatment had five replicates and plants were harvested after 40 days.

### Experiment 2: how common is the AMF suppression?

Prevalence of, and variation in, the AMF-suppressive effects of field soil was studied using 21 Scandinavian field soils. All soils were labelled with 3 kBq g^−1^ of carrier-free H_3_^33^PO_4_ that was uniformly mixed into the soil before packing 55 g soil into a HC. HCs of each soil, replicated four times, were buried into pots containing semi-sterile standard soil amended with 10 mg P per kg and mixed with 75 g of *R. irregularis* inoculum (total weight: 1.0 kg). Two *M. truncatula* plants were grown in each pot, and harvested after 32 days.

### Experiment 3: what are the AMF-suppressive components of field soils?

Two soils, Møystad E2 and Risø stored, had contrasting AMF-suppressive effects in Expt. 2 and were thus selected for further investigation into the AMF-suppressive components of field soils. To determine whether soil pH affects soil suppressive activity, the strongly AMF-suppressive Møystad E2 soil, which had a baseline pH of 4.4, was supplemented with CaCO_3_ at the rates of 0.25, 1.0 and 4.0 g of CaCO_3 _per kg soil to achieve new soil pH levels of 4.7, 5.5 and 7.1, respectively. To address whether AMF suppression has a biological background, the two soils were adjusted to 10 % water and subjected to a pasteurisation in a water bath (85 °C, 90 min). Corresponding non-pasteurised treatments were included as controls. To distinguish between general or specific AMF suppression, a soil transfer experiment was set-up as follows. Two soils deemed ‘conducive’ from Expt. 2 (Møystad E7 and Risø stored) were mixed in the following ratios with the respective ‘suppressive’ partner soil (Møystad E2 and Risø): 10:0; 9:1; 1:1; 1:9; and 0:10. Following these modifications, the soils were mixed in a 1:1 ratio with sand, labelled with radioisotope at 4 kBq g^−1^ soil carrier-free H_3_^33^PO_4_ and packed into HCs. Pots and seeds were prepared as for Expt. 2, with three replicates per treatment. Plants were harvested after 29 days.

### Harvesting and sample analyses

Plants were harvested and analysed as follows: roots were separated from shoots; roots were washed free of soil; blotted dry; and a weighed subsample was stored in 50% EtOH for determination of mycorrhizal root colonisation by microscopy after clearing in 10% KOH and staining with Trypan blue (modified from ref. [33]). The remaining root tissue and the total shoot tissue was dried at 70 °C (>48 h) and dry weights recorded.

Shoots were ground before acid digestion, and analysis of the shoot ^33^P and ^32^P contents was conducted using a Packard liquid scintillation counter (PerkinElmer, Waltham MA, USA). Subsamples of soil from each HC were taken for quantification of soil P concentration, soil pH, AMF hyphal length density (HLD; Expt. 1 only), phospholipid-derived fatty acid (PLFA) analysis (Expt. 3) and 16S rRNA gene sequencing (Expt. 2 and 3). HLD was determined in duplicates of 2 g dry soil by a grid intersect method [34]. Soil P status was determined from P concentrations in extracts with water [35] or NaHCO_3_ [36], and soil pH was determined after shaking with CaCl_2_ [37].

PLFAs were extracted from 2 g frozen soil according to ref. [38] using nonadecanoic acid as the internal standard. Samples were analysed by gas chromatography mass spectromety using aVarian CP482fitted with a 50 m CPSIL8CB column (Ø 0.25 mm, film thickness 0.25 µm), after injection of 3 µL using a temperature programme of 50 °C for 1.89 min, 20 °C min^−1^ to 160 °C, hold for 5 min, 5 °C min^−1^ to 270 °C, hold for 0 min, 50 °C min^−1^ to 325 °C, hold for 3 min. The mass spectometer was operated in a scan mode, *m*/*z* 50–300.

DNA was isolated from frozen HC soil by bead-beating and phenol-chloroform extraction as described in ref. [39]. Each DNA sample contained pooled DNA isolated from two subsamples of 0.5 g soil from each HC. Subsequently, 16S rRNA gene copies were quantified by quantitative PCR (qPCR) using primers bac341F and bac907R [40, 41]. Reactions were carried out with 1× Brilliant III SYBR Green QPCR Master Mix (Agilent Technologies, USA), 0.4 µM of each primer and 1 mg ml^−1^ bovine serum albumin under thermal cycling conditions: 95 °C for 3 min followed by 35 cycles of 95 °C for 15 s and 57 °C for 20 s.

Illumina MiSeq 300 bp paired-end sequencing of the hypervariable V3-V4 regions of the 16S rRNA gene, amplified with primers Bakt_341F and Bakt_805R (from ref. [42]) was performed on the extracted DNA by Macrogen Inc. (Seoul, Rep. of Korea). Sequencing of soil DNA from Expt. 2 and Expt. 3 was done in two separate sequencing runs and data were analysed separately. Sequencing data were analysed using the CLC Genomics Workbench with the Microbial Genomics Module (Qiagen) using the software’s default settings. Partial 16S rRNA gene sequences were clustered and assigned to operational taxonomic units (OTUs) with 97% similarity using the SILVA 16S rRNA reference database release 119 without de novo OTU clustering.

### Statistics

A two-way analysis of variance (ANOVA) was conducted on Expt. 1 data for each HC (semi-sterile or unsterile) separately, with the factors (1) soil in rooting compartment (semi-sterile or unsterile) and (2) AMF inoculation (inoculated with *F. mosseae* or not). Shoot ^33^P content, ^32^P content and HLD were analysed by Student’s *t*-test to compare between the two HCs within each treatment.

One-way ANOVA compared the shoot ^33^P activity in plants grown across 21 unsterile soils (Expt. 2), in Møystad E2 soil of increasing pH (Expt. 3) and in the soil dilution experiment for the pairs of Møystad and Risø soils, respectively (Expt. 3), with Tukey’s post hoc tests conducted where appropriate (JMP Pro v. 12.0.1). For the pasteurisation experiment, Student’s *t*-tests were used to compare shoot ^33^P content from soils with and without pasteurisation treatment.

Regression analyses were performed between shoot radioactive P content and HLD (Expt. 1), and between shoot ^33^P content and a number of HC soil physicochemical characteristics (Expt. 2). Data that were non-normal, including shoot ^33^P content (as per Shapiro–Wilk test), were subjected to a log-transformation prior to statistical analyses. Statistical analyses were performed using JMP Pro v. 12.0.1, except PLFA data that were normalised and analysed by principal components analysis using Minitab v. 17.

For the purpose of the 16S rRNA gene amplicon data analyses, soils were considered AMF-suppressive when plant ^33^P uptake <1 kBq per pot. Principal coordinates analyses (PCoAs) were performed on Bray–Curtis dissimilarity matrices originating from 97% nucleotide similarity OTU tables using the software PAST v.2.17. Statistical significance of the overall difference in microbial community composition was tested by PERMANOVA analysis on Bray–Curtis dissimilarities with 9999 permutations using the Adonis function of the vegan package implemented in R. The α-diversity, represented by Shannon H and Chao-1 estimations, was calculated in PAST and compared by Student’s *t*-test. The online tool Venny (http://bioinfogp.cnb.csic.es/tools/venny/index.html) was used for Venn diagram construction. Similarity percentage analysis (SIMPER) on Bray–Curtis dissimilarities of genus-level OTU tables was performed in PAST. Differences in taxa abundance between the soil groups were tested by one-way ANOVA, with Tukey’s post hoc test using STAMP v. 2.1.3. Filtered genus-level OTU tables and the environmental variables pH, SOM, P, clay, silt and sand (Table S1) were included in a canonical correspondence analysis (CCA) performed in PAST, and significant correlations between the variables were tested by Spearman correlation analysis on the full genus-level OTU table in PAST. 16S rRNA gene amplicon sequences are deposited in NCBI’s Sequence Read Archive under BioProject PRJNA408226.

## Results

### Do field soils suppress production and activity of ERM?

**Fig. 1:**
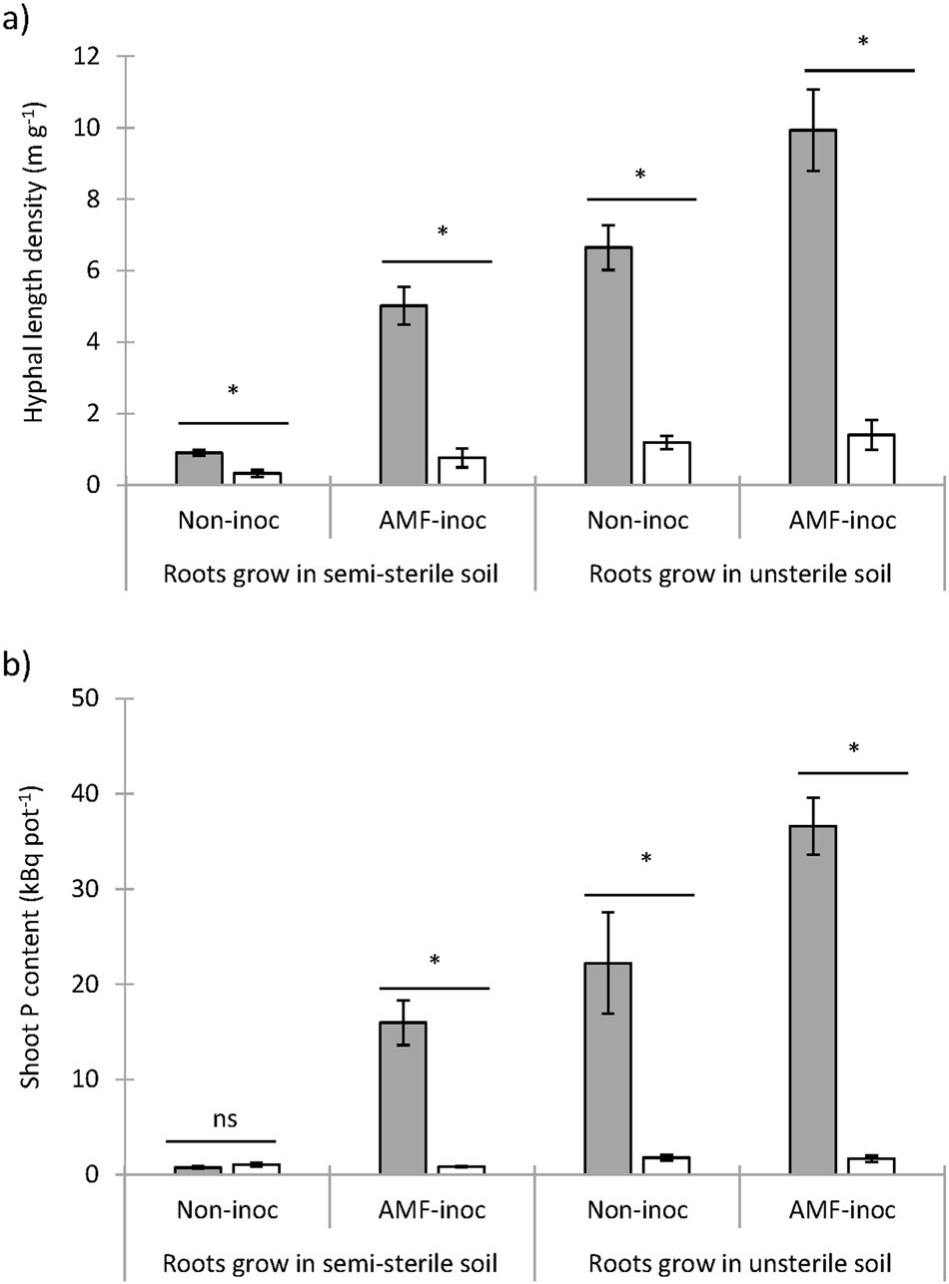
AMF hyphal length density (**a**) in the hyphal compartments (HCs), and shoot ^33^P and ^32^P contents (**b**) taken up from the HCs containing either semi-sterile (^33^P; grey bars) or unsterile (^32^P; white bars) Risø soil. The AMF mycelium was derived from the inoculant fungus (*F. mosseae*) in semi-sterile soil, or from the native AMF community/inoculant *F. mosseae* in unsterile Risø soil (Expt. 1)

Roots growing in soil with AMF propagules in Expt. 1 were abundantly colonised by the fungi (66% colonised root length in inoculated semi-sterile soil; 65% in unsterile soil; and 68% in inoculated unsterile soil). HLD was significantly (*P* < 0.05) lower in the HC with unsterile than in the HC with semi-sterile soil (Fig. 1a), and radioisotope uptake into shoots was significantly lower from unsterile soil (^32^P) than from semi-sterile soil (^33^P) (Fig. 1b). Plants grown in the absence of AMF propagules in semi-sterile soil remained non-colonised, and the corresponding hyphal lengths and uptake of radioisotopes were at background levels. Regression analysis between shoot radioactive P content and HLD in the relevant HC revealed a significant (*P* < 0.0001) and strong, positive (*R*^2^ = 0.91) relationship. This experiment demonstrated that natural soil in the HC can inhibit growth of, and P uptake by, ERM. The high correlation between hyphal length and mycorrhizal radioisotope P uptake in shoots allowed for the latter measure to be used as a proxy for ERM activity in subsequent experiments.

### How common is the AMF suppression?

**Fig. 2:**
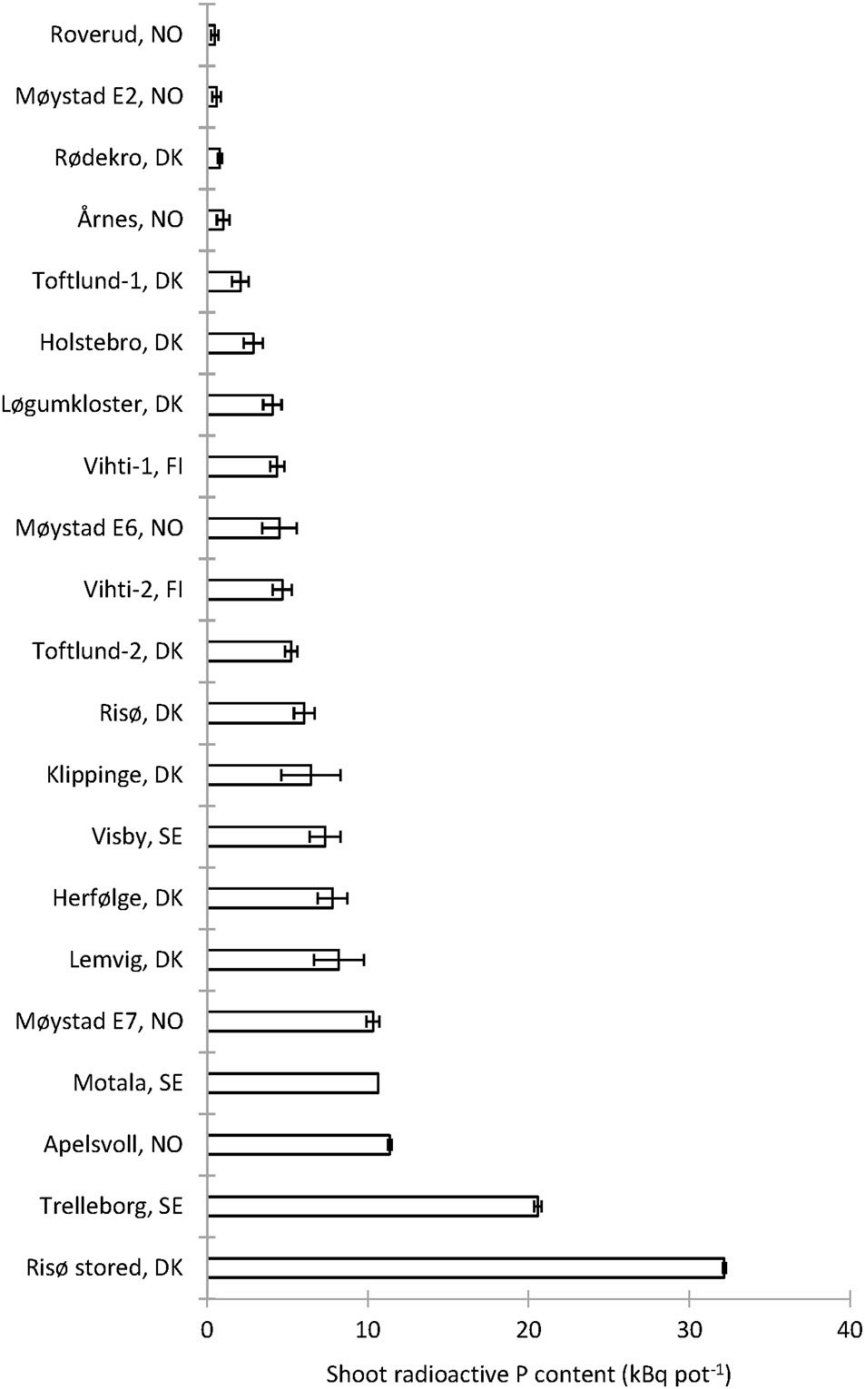
Shoot uptake of ^33^P by AMF from HCs containing 1 of 21 different unsterile soils (two plants per pot). Root external mycelium grew from *M. truncatula* roots colonised by *R. irregularis* (Expt. 2)

The P-transfer activity of AMF was subsequently determined in an experiment where the HC contained 1 of 21 unsterile soils with contrasting properties (Table S1). Root colonisation as a result of AMF inoculation was high with little variation across treatments (86 ± 0.9%), and all HCs thus had the same potential for ERM ingrowth. However, hyphal transport of ^33^P to shoots varied greatly across the 21 unsterile soils (*P* < 0.0001; see Table S2 for ANOVA outcomes). Four soils, Roverud, Møystad E2, Rødekro and Årnes, were AMF-suppressive, while Risø stored and Trelleborg were the most AMF-conducive soils (Fig. 2). Furthermore, the Risø stored soil conferred significantly more ^33^P to the plant than any other soil tested. A significant (*P* < 0.0001), positive correlation (*R*^2^ = 0.493) was found between pH of the HC soil and shoot ^33^P content (Figure S2a). No other significant correlations were found between ERM activity and physicochemical properties of the soils; this includes a lack of correlation with water-extractable P (Figure S2b). Overall, these results demonstrated that the ERM activity was highly soil-dependent, and highly suppressed in four soils.

### What causes AMF suppression in field soils?

**Fig. 3:**
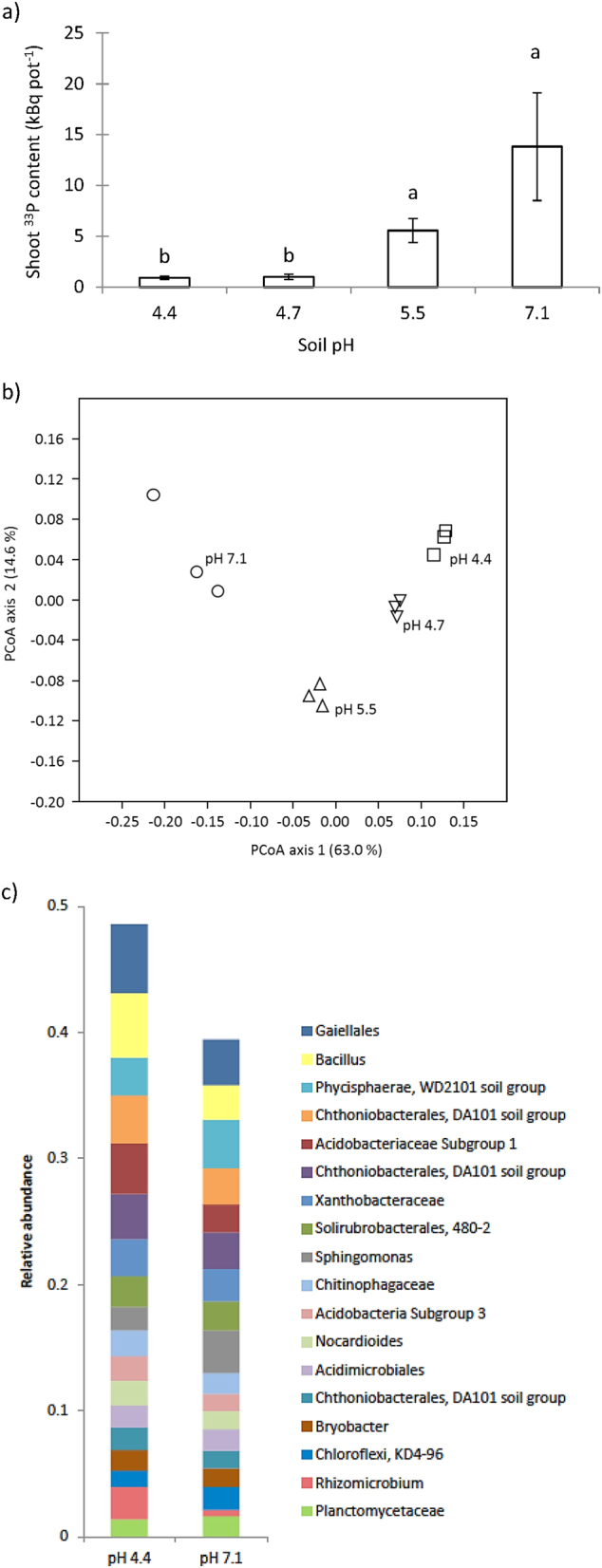
Shoot ^33^P uptake (**a**) and principal coordinates analysis (PCoA) of bacterial communities (**b**) from the HC containing unsterile Møystad E2 soil, amended with increasing amounts of CaCO_3_ to increase soil pH (Expt. 3). The PCoA was made on Bray–Curtis dissimilarities of OTUs with 97% similarity. The relative abundance of genus- or higher-level taxa (**c**) in Møystad E2 soils with pH 4.4 or 7.1 (Expt. 3). Only taxa constituting >1.5% of total abundance in the two soils are included in the bar plot

**Fig. 4:**
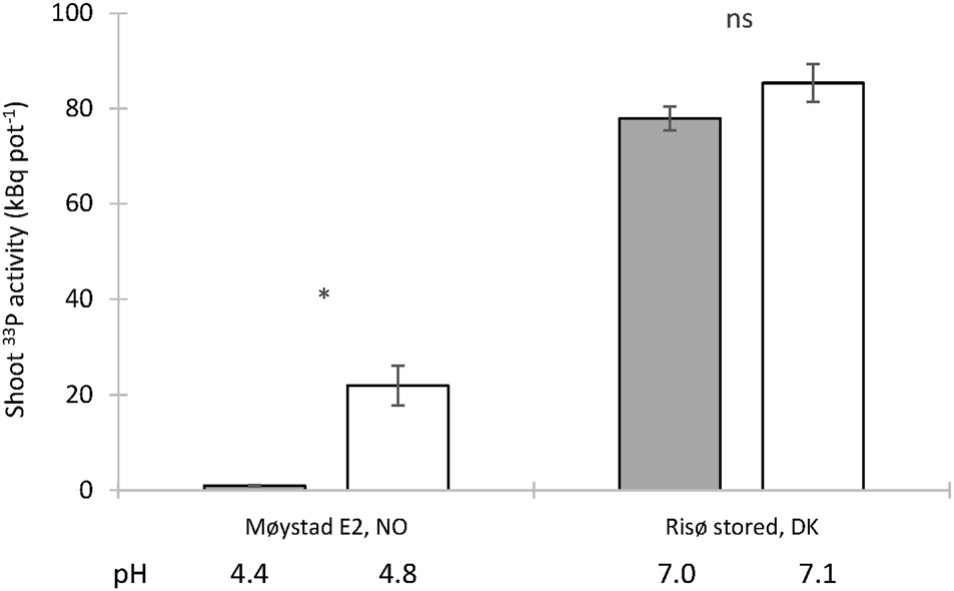
Shoot ^33^P uptake from HCs containing one of two unsterile soils (one AMF-suppressive and one AMF-conducive), either pasteurised by heating at 80 °C (white bars) or not heated (grey bars) (Expt. 3)

**Fig. 5:**
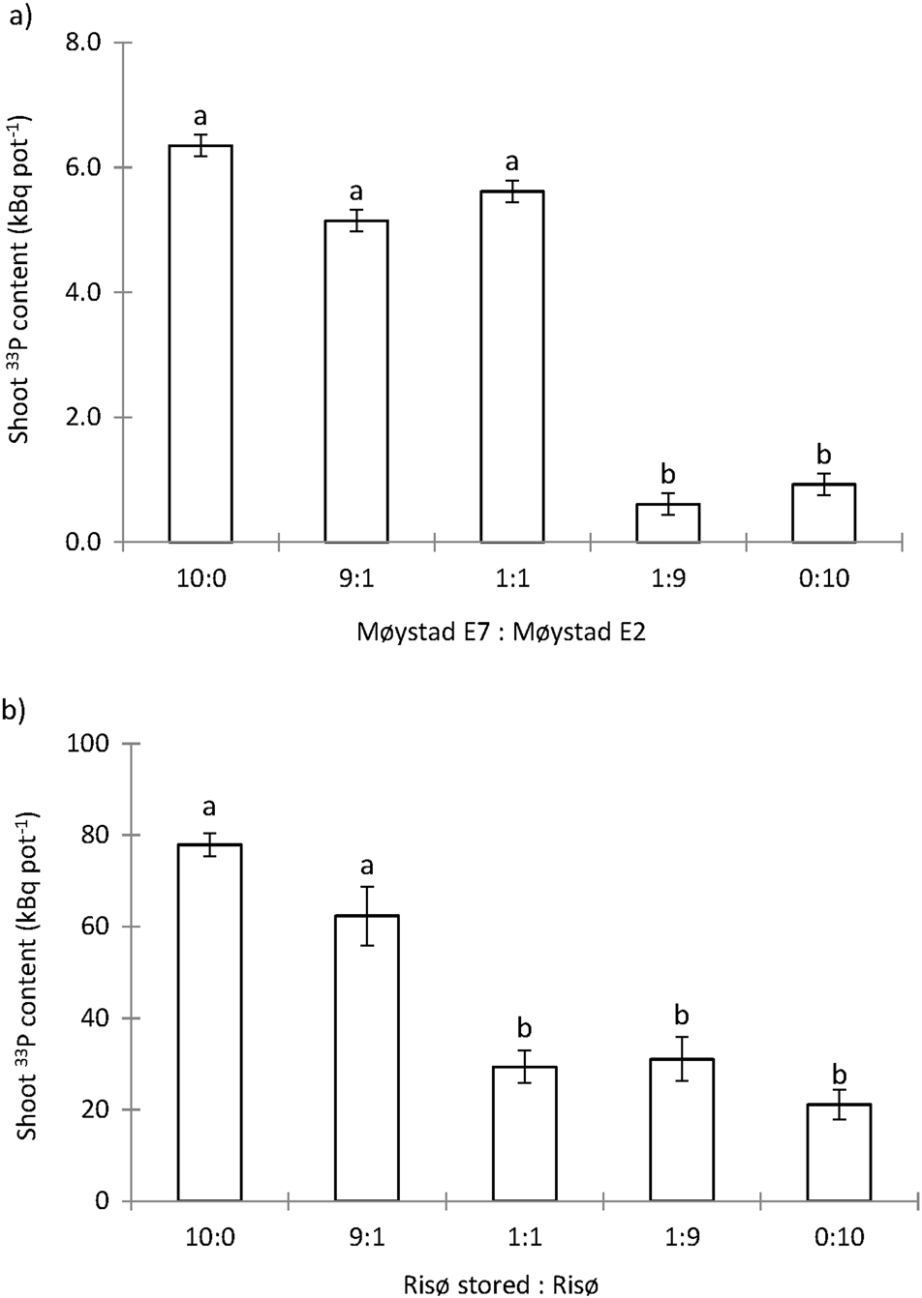
Shoot ^33^P uptake from HCs containing **(a)** either Møystad E2 or Møystad E7 soil, or three different mixes of the two soils and (**b)** either Risø stored or Risø soil, or three different mixtures of the two soils. Root external mycelium grew from *M. truncatula* roots colonised by *R. irregularis* (Expt. 3)

Hyphal transport of ^33^P from the AMF-suppressive Møystad E2 soil increased when soil pH was increased from 4.4 to 7.1 (*P* < 0.001; Fig. 3a, Table S2). In addition, pasteurisation of this soil led to significantly higher values of shoot ^33^P content than were obtained from the non-pasteurised soil (Fig. 4). Additionally, the suppression of ERM activity was mitigated by pasteurisation in two other soils (data not presented), and by irradiation of Risø soil (Fig. 1). In contrast, pasteurisation of the AMF-conducive soil (Risø stored) did not lead to increased shoot ^33^P content (Fig. 4). These experiments showed that besides a pH effect, suppression had a biological component. Soil transplantation experiments prepared with two pairs of conducive vs. suppressive soils showed that significant suppression required at least a 1:1 mixing of suppressive with conducive soil (Fig. 5).

Principal component analysis of microbial PLFAs from Møystad E2 soil with different pH showed clear pH-induced changes in the bacterial community (Figure S3). Notably, five non-saturated iso- and antheiso PLFAs (*r* = −0.59 to −0.73; 0.04 > *P* > 0.01), which are markers for Gram-positive bacteria, correlated negatively with pH. Similarly, PCoA of 16S rRNA gene sequences at 97% similarity OTU level of the same soils showed a shift in bacterial community composition along the PCoA axis 1 (explaining 63.0% of the variation) in response to increasing soil pH (Fig. 3b). Species richness, estimated by Chao-1, was significantly (*P* < 0.05) higher in soil limed to pH 7.1 than in the untreated soil at pH 4.4, but no significant change was observed in the Shannon H-index (Figure S4a). The taxa that contributed to most of the variation between soils at pH 4.4 and 7.1 were identified by SIMPER. The largest decreases in relative abundance of the taxa being significantly influenced by liming (*P* < 0.05) were exhibited by Acidobacteriaceae subgroup 1 (Gp1), *Bacillus*, *Rhizomicrobium* and *Gaiellales* (Fig. 3c).

**Fig. 6:**
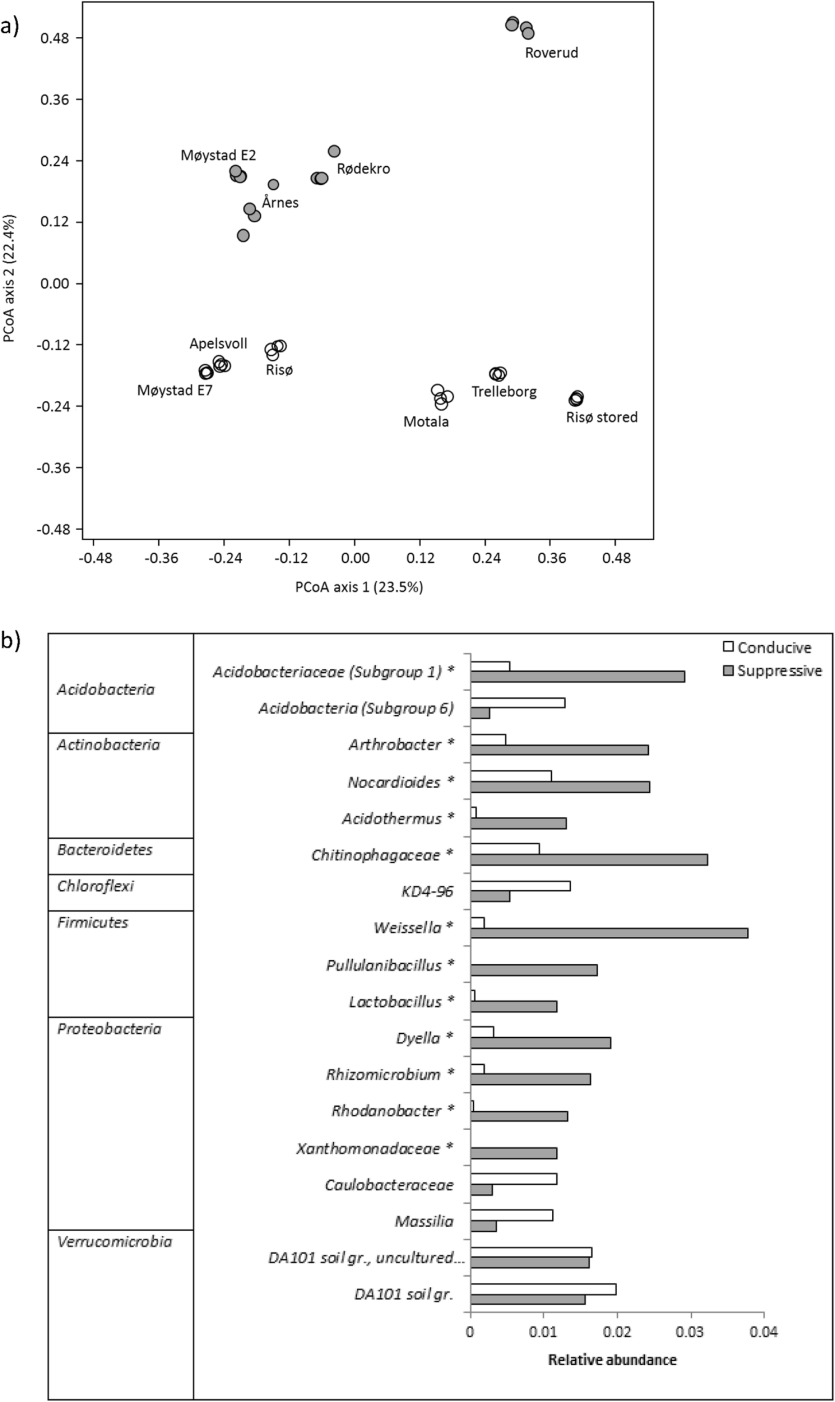
Principal coordinate analysis (**a**) of bacterial communities in soils suppressive (filled circles) for AMF and in non-suppressive (open circles) soils (Expt. 2). Suppressive soils are defined as the soils where plant ^33^P uptake was <1 kBq per pot. The analysis was made on Bray–Curtis dissimilarities between OTUs with 97% similarity. Relative abundances (**b**) of genus- or higher-level taxa that contribute to most of the variation between the bacterial communities in AMF-suppressive and non-suppressive soils identified by SIMPER analysis and are present in significantly different abundances in suppressive and conducive soils (Expt. 2). Taxa marked with an asterisk (*) were observed in significantly (ANOVA; *P < *0.05) higher abundance in AMF-suppressive soils than in conducive soils

Subsequently, 16S rRNA gene-targeted analyses were used to investigate whether specific bacterial groups were associated with AMF suppression in a broader range of soils (Fig. 2); four highly AMF-suppressive (plant ^33^P uptake < 1 kBq per pot) soils, and six soils that conferred intermediate to high plant ^33^P uptake. Interestingly, PCoA at the 97% similarity OTU level revealed clear clustering of AMF-suppressive vs. AMF-conducive soils along the PCoA axis 2, explaining 22.4% of the variation (Fig. 6a). Furthermore, three of the AMF-suppressive soils clearly clustered together, while the Roverud soil diverged from the other suppressive soils on both PCoA axes. The six AMF-conducive soils clustered into two groups, mostly along PCoA axis 1. Separation of the AMF-suppressive and AMF-conducive soils was tested by PERMANOVA analysis with Bonferroni correction, which showed a significant separation (*P* < 0.05) up to phylum level (Table S3). The separation of AMF-suppressive and AMF-conducive soils was connected neither to differences in 16S rRNA gene copy number (data not shown) nor to differences in α-diversity as determined by species richness (Chao-1) and diversity (Shannon H-index; *P* > 0.05; Figure S4b). Nevertheless, as also indicated by rarefaction curves (Figure S5), diversity was distinctly lower in the divergent AMF-suppressive Roverud soil, when compared with the remaining suppressive soils, and distinctly higher in the cluster of AMF-conducive soils from Møystad E7, Apelsvoll and Risø, when compared with the three other conducive soils that clustered together in the PCoA plot.

**Fig. 7:**
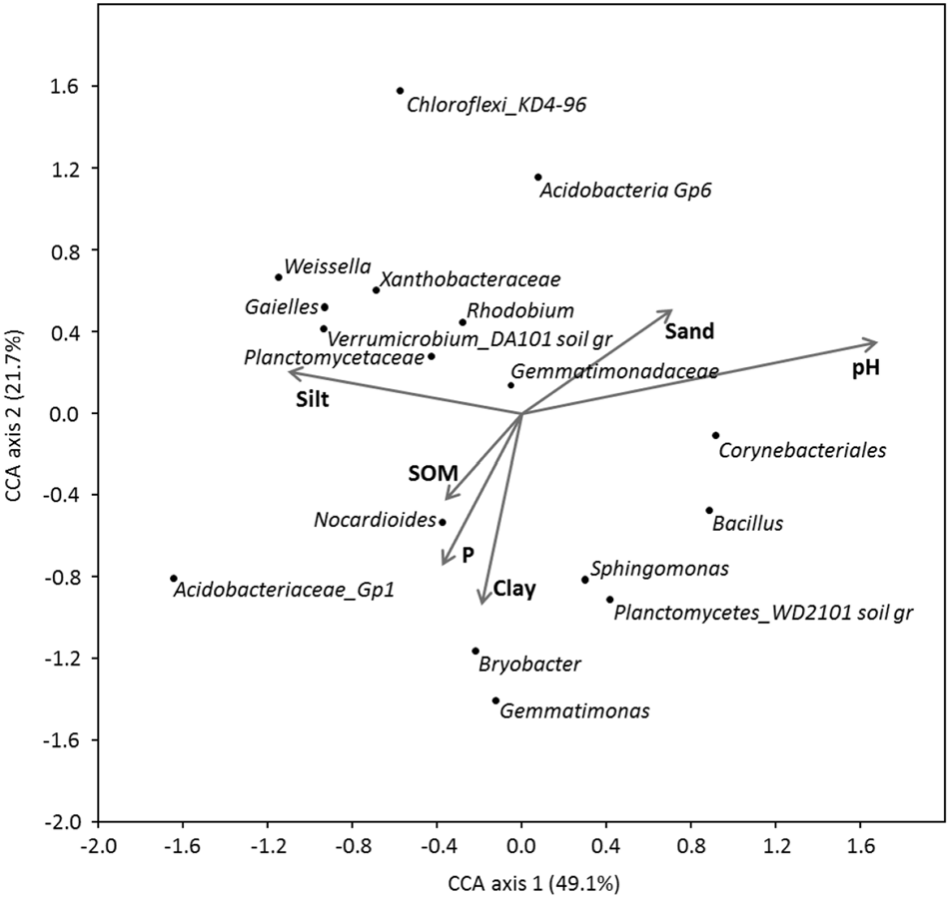
Canonical correspondence analysis (CCA) between genus- or higher-level relative abundance and environmental variables (Expt. 2). Only taxa contributing to >1 % relative abundance of all taxa are included in the plot

Only a small proportion (3%) of the total number of bacterial genera-level OTUs was unique to the AMF-suppressive soils, while the majority (82%) of the genera was shared between the suppressive and conducive soils (see Venn diagram, Figure S6). The most abundant of the genera unique to the suppressive soils had a relative abundance of 0.02 % (Table S4), and the remaining unique genera were even less abundant. This suggests that AMF suppression is coupled with a higher relative abundance of one or more taxa. To identify such taxa, a SIMPER analysis was performed. The taxa that contributed most to the variation (cumulative 40%) between suppressive and conducive soils, and that were present in significantly different relative abundances in the two groups of soils, are presented in Fig. 6b. Of these, taxa belonging to Acidobacteriaceae Gp1, *Acidothermus*, *Arthrobacter*, *Norcardioides*, Chitinophagaeceae, *Lactobacillus*, *Pullanibacillus*, *Weissella*, *Dyella*, *Rhizomicrobium*, *Rhodanobacter* and Xanthomonadaceae were significantly (ANOVA; *P* < 0.05%) more abundant in AMF-suppressive than in AMF-conducive soils. The largest differences in relative abundances were found for *Weissella* and *Acidobacteriaceae* Gp1. Furthermore, CCA (Fig. 7) and regression analysis revealed that the relative abundance of *Acidobacteriaceae* Gp1 was significantly (*P* < 0.01), negatively, correlated to soil pH. No other taxa identified by SIMPER analysis correlated significantly with any of the measured soil physicochemical properties.

## Discussion

This is the first systematic study to show that the activity of AMF mycelium (ERM) is inhibited in natural soil, and that the degree of suppression varies between soils. This suppression resembles previous observations made of disease-suppressive soils that minimise plant diseases caused by pathogenic fungi. While disease suppression in soil is often associated with specific groups or consortia of microorganisms [29, 30], our study suggests that suppression of AMF involves both abiotic and biotic components. Importantly, suppression was observed with ERM growing from roots colonised by each of two individual AMF isolates: *F. mosseae* and *R. irregularis*, and also with a native AMF field community. The results hence suggest that soil-associated suppression of ERM activity occurs across different AMF taxa.

The suppression of ERM activity is a bulk soil phenomenon as the observed effects were exerted on hyphae >0.5–1 mm from the root surface (being the approx. extension of root hairs penetrating the HC meshes). Yet, AMF colonisation of roots was not suppressed by a soil that was otherwise suppressive to ERM activity.

While plant P sufficiency is known to suppress AMF colonisation of roots [43] and soil [44], and thereby the symbiotic C costs, plants grown at sub-optimal P conditions will depend on AMF for maintaining P sufficiency. In the current experiments, soil suppressiveness did not correlate to water-extractable soil P. Likewise, the specific activity of ^33^P in aqueous extracts of HC soil was rather constant across soils that were suppressive and conducive and decreased only little with increasing soil P (data not shown). Thus, reduced uptake of ^33^P in some soils is not likely to have resulted from competition for ^33^P with soil microbes. ERM suppression could interfere with a range of soil processes that are stimulated by ERM: uptake of P and other nutrients [45–48]; distribution of plant C to bulk soil bacteria [49]; soil aggregate formation [50]; SOM mineralisation [51]; and formation of mycorrhizal networks [52].

The reduced suppression of ERM activity in semi-sterile soils shows that suppression involves a biotic component. For comparison, reduction of suppression by steaming, pasteurisation or fumigation is a key property used to define disease-suppressive soils [23]. Further, the observation that suppression against AMF could not be transferred by adding small amounts of suppressive soil to conducive soil indicates that the suppression of AMF is so-called general suppression rather than a specific suppression, for which transferability is a hallmark [23].

Soil pasteurisation mitigated AMF suppression much more than liming treatments, and the biotic component therefore dominated over an abiotic pH effect. The observed weak correlation between pH and ERM activity among the 21 field soils agrees with reports that low pH is suppressive to the Glomeracea, possibly via a direct effect on growth [25, 53]. However, the composition of the bacterial microbiome is strongly pH-dependent [54]; here 16S rRNA gene sequencing and PLFA analysis indeed showed a clear effect of liming on the bacterial community composition in a selected soil. Hence, the observed pH effect on ERM activity may well work via effects on ERM-suppressive bacteria. We found significantly higher abundance of Acidobacteria, Gp1, *Bacillus*, *Rhizomicrobium* and *Gaiellales* in un-limed soil at pH 4.4 than in limed soil at pH 7.1. For the Acidobacteria Gp1, a broader analysis of 10 different soils confirmed their higher abundance at low pH, in agreement with previous observations [54, 55]. Although the correlation of Acidobacteria Gp1 abundance with pH may confound a direct correlation with suppression, OTUs belonging to this group are frequently associated with disease-suppressive soil. For instance, they are observed in higher frequencies in the rhizosphere outside patches of *Rhizoctonia*-diseased plants, and in soil suppressive to *Fusarium* wilt or take-all disease caused by *Gaeumannomyces* [1, 56, 57].

*Bacillus* was also significantly more prevalent in the Møystad E2 soil at pH 4.4 than at pH 7.1, but its abundance did not correlate to pH in the broader analysis of 10 soils. *Bacillus* is well-known for its antifungal properties and high abundance in soils suppressive for fungal diseases [22, 58–60]. As *Bacillus* produces heat-resistant endospores [61], the lower suppression in the Møystad E2 soil after pasteurisation suggests that AMF suppression in this soil could not be explained by the abundance of *Bacillus* alone. *Rhizomicrobium* and *Gaiellales* have not, to our knowledge, been associated with suppressive soils.

As general suppression is considered to be caused by the collective competitive and antagonistic effects of the soil microbial community [21], we compared the microbiomes of 10 selected AMF-suppressive or AMF-conducive soils to identify additional groups potentially contributing to the suppression. We found significant differences in community composition between the soils defined as suppressive or conducive; suppressive soils had increased abundance of not only Acidobacteria Gp1, but also several indicator taxa, primarily affiliated with the Actinobacteria, Bacteriodetes, Firmicutes and Proteobacteria. Members of these groups have repeatedly been associated with the suppression of plant diseases [59, 62, 63]. Although some studies found few specific groups dominating in disease-suppressive soils [60, 64], the relatively high number of indicator taxa in the current study supports the hypothesis that AMF suppression results from the interactive effects of several microbial groups, as found for some disease-suppressive soils [59, 65, 66]. Indeed, putative fungal antagonists were most abundant in the AMF-suppressive soils; in addition to Acidobacteria Gp1, Firmicutes OTUs were particularly more abundant. Among these, *Weissella* can be abundant in *Fusarium*-suppressive soil [67], and both *Weissella* and *Lactobacillus* are antagonistic to several fungi in vitro [68–70].

Production of antimicrobial metabolites, even organic acids, may be involved in a putative antagonism. The Actinobacteria OTUs with increased abundance in the AMF-suppressive soils included OTUs from the genera *Acidothermus*,* Nocardioides* and *Arthobacter*. Actinobacteria are recognised for their ability to produce an array of antibiotics and enzymes [71] that may play a role in antagonistic interactions with other soil microorganisms. Members of the family Chitinophagaceae produce antifungal metabolites when co-cultured with other bacteria [72], and may associate with the decomposing mycelia of ectomycorrhizal fungi [73]. From the Proteobacteria, OTUs affiliated with two genera of the Xanthomonaceae, *Rhodanobacter* and *Dyella*, were far more abundant in AMF-suppressive soils. *Rhodanobacter* has previously been associated with the suppression of compost-amended potting mix, and isolates show antagonism against a root-rot pathogen [74]. *Dyella* was more abundant in soil patches that had recovered from *Rhizoctonia* bare patch disease [57], but without strong correlation to suppression.

### Conclusions and wider perspectives

Soils that suppress ERM P uptake activity appear to be abundant. Suppression involves both an abiotic (pH) component, and a biotic component related to so-called general suppression. Hence, the mechanisms behind suppression of ERM activity are most likely due to the combined action of several members of the soil microbial communities, and our study points to several taxa that could play a role in AMF suppression. Further studies need to isolate the candidate suppressive bacteria and perform manipulation experiments (with various bacterial isolates, alone or in combination) to test their suppressive effects on foraging activities by ERM. Future studies should also consider other soil microbiome members with potential suppressive effects such as fungal taxa, and aim to elicit the specific antagonistic or competitive mechanisms involved. This study emphasises that extrapolations to field conditions from AMF model systems using semi-sterile soil could be misleading, and that the ecosystem services of the AMF community will depend strongly on the specific soil microbiome.

## Electronic supplementary material

Supplementary information
