# Supplementary material for: Suppression of the activity of arbuscular mycorrhizal fungi by the soil microbiota
Source: ISME J. 2018 Jan 30;12(5):1296–307. doi: 10.1038/s41396-018-0059-3 (PMC5931975; doi:10.1038/s41396-018-0059-3)
Supplement: Supplementary file 1 — Supplementary information [file 41396_2018_59_MOESM1_ESM.docx]

Supplementary information to Svenningsen et al.:

Table S1: Geographic and physicochemical details of the various Scandinavian soils used in the hyphal compartments (HCs) for the experiments.

| **Soil name** | **Country of origin** | **Coordinates N** | **Coordinates E** | **pH (CaCl_2_)** | **SOM (%)** | **Clay** | **Silt** | **Sand** | **Olsen P (mg/100g)** |
| --- | --- | --- | --- | --- | --- | --- | --- | --- | --- |
| Herfølge | DK | 55°25' | 12°06' | 7.0 | 2.5 | 15 | 15 | 68 | 1.2 |
| Holstebro | DK | 56°22' | 8°45' | 5.7 | 3 | 4 | 3 | 90 | 5.3 |
| Klippinge | DK | 55°20' | 12°18' | 6.8 | 2.7 | 19 | 14 | 65 | 1.4 |
| Lemvig | DK | 56°36' | 8°13' | 5.1 | 2.2 | 4 | 2 | 91 | 2.6 |
| Løgumkloster | DK | 55°06' | 9°02' | 5.3 | 32 | nd | nd | nd | 2.2 |
| Risø stored | DK | 55°41' | 12°06' | 7.1 | 2.1 | 11 | 13 | 74 | 1.2 |
| Risø | DK | 55°41' | 12°06' | 5.5 | 2.1 | 10 | 12 | 76 | 1.1 |
| Rødekro | DK | 55°03' | 9°13' | 5.0 | 10.3 | 10 | 6 | 73 | 5.3 |
| Toftlund-1 | DK | 55°09' | 9°05' | 5.3 | 5.2 | 6 | 5 | 85 | 5.3 |
| Toftlund-2 | DK | 55°09' | 9°04' | 5.8 | 5.5 | 5 | 5 | 84 | 4.1 |
| Vihti-1 | FI | 60°21' | 24°22' | 5.9 | 4.5 | 61 | 20 | 15 | 2.2 |
| Vihti-2 | FI | 60°22' | 24°23' | 6.0 | 4.6 | 45 | 33 | 18 | 3.3 |
| Apelsvoll | NO | 60°42' | 10°52' | 5.6 | 7.2 | 18 | 35 | 47 | 2.4 |
| Årnes | NO | 60°10' | 11°30' | 5.1 | 3.5 | 33 | 35 | 29 | 2.7 |
| Møystad E2 | NO | 60°56' | 11°37' | 4.4 | 5.0 | 16 | 37 | 47 | 6.8 |
| Møystad E6 | NO | 60°56' | 11°37' | 5.4 | 6.7 | 16 | 37 | 47 | 1.9 |
| Møystad E7 | NO | 60°56' | 11°37' | 5.6 | 8.2 | 16 | 37 | 47 | 4.0 |
| Roverud | NO | 60°15' | 12°05' | 5.1 | 1.8 | 8 | 31 | 60 | 3.2 |
| Motala | SE | 58°33' | 15°10' | 5.9 | 5.8 | 41 | 39 | 14 | 1.7 |
| Trelleborg | SE | 55°24' | 13°02' | 5.8 | 2.2 | 12 | 14 | 72 | 0.9 |
| Visby | SE | 57°34' | 18°24' | 6.6 | 3 | 17 | 29 | 50 | 1.2 |

Table S2. Summary of ANOVA outcomes for relevant response variables.

|  |  | **Soil sterilisation** | **AMF inoculation** | **S * A** |
| --- | --- | --- | --- | --- |
| Expt. 1 | Shoot P from unsterile HC | 0.0042 | ns | ns |
|  | Shoot P from semi-sterile HC | <0.0001 | <0.0001 | <0.0001 |
|  | HLD from unsterile HC | ns | ns | ns |
|  | HLD from semi-sterile HC | <0.0001 | <0.0001 | <0.0001 |
|  |  |  |  |  |
|  |  | **Soil location** |  |  |
| Expt. 2 | Shoot P from HC | <0.0001 |  |  |
|  |  |  |  |  |
|  |  | **Soil pH** |  |  |
| Expt. 3 | Shoot P from HC | 0.0004 |  |  |
|  |  | **Soil dilution** |  |  |
| Expt. 3 | Shoot P from HC - Møystad | <0.0001 |  |  |
|  | Shoot P from HC - Risø | <0.0001 |  |  |

Factors in the analysis are indicated in bold text. The main effects and interaction term are indicated where relevant. *P*<0.05 was considered a significant outcome. “ns” indicates a not significant outcome.

Table S3. PERMANOVA analysis assessing the community differences at four taxonomic levels after grouping of soils into suppressive and conducive (Expt. 2). The analysis was performed on Bray-Curtis dissimilarities. Bonferroni corrected *p*-values are shown.

| **Grouping** | **97% OTU, *p*-value (Bonferroni corr.)** | **Family, *p*-value**  **(Bonferroni corr.)** | **Class, *p*-value (Bonferroni corr.)** | **Phylum, *p*-value (Bonferroni corr.)** |
| --- | --- | --- | --- | --- |
| AMF suppressive vs. non-suppressive | 0.0001 | 0.0001 | 0.0021 | > 0.05 |

Table S4. Relative abundance of genera or higher order taxa only found in the suppressive soils.

| **Taxa** | **Relative abundance (%)** |  | **Taxa** | **Relative abundance (%)** |
| --- | --- | --- | --- | --- |
| Candidatus Methylacidiphilum | 0.0216 |  | Amphibacillus | 0.00025 |
| Xanthomonadales, uncultured | 0.01166 |  | Lactobacillales, uncultured bacterium | 0.00025 |
| Candidate division TM7 bacterium LY2 | 0.01026 |  | Gordonibacter | 0.00025 |
| Candidate division SAM bacterium | 0.00896 |  | Coprobacter | 0.00025 |
| Humibacter | 0.00127 |  | Porphyromonadaceae, uncultured | 0.00025 |
| Marisediminicola | 0.00112 |  | Weeksella | 0.00025 |
| Armatimonadales, uncultured soil bacterium | 0.000598 |  | Muricauda | 0.00025 |
| Moraxella | 0.000498 |  | Nitrospiraceae, uncultured | 0.00025 |
| Rhodospirillales, uncultured bacterium | 0.00049 |  | Chroococcidiopsis | 0.00025 |
| Thermomicrobia, uncultured | 0.000398 |  | Caldisericales, uncultured bacterium | 0.00025 |
| Parvimonas | 0.000374 |  | Candidatus Liberibacter | 0.000199 |
| Alloscardovia | 0.000374 |  | Candidimonas | 0.000199 |
| Candidate division TM7, uncultured | 0.000374 |  | Acidobacteria Subgroup 13, uncultured Acidobacteria bacterium | 0.000199 |
| Acidobacteria Subgroup 15, uncultured Acidobacteria bacterium | 0.000298 |  | Dethiobacter | 0.000196 |
| Sneathiellaceae, uncultured | 0.000294 |  | Uncultured Bacillus sp. | 0.000196 |
| Thermomonosporaceae, uncultured | 0.000294 |  | Ktedonobacteria, uncultured bacterium | 0.000196 |
| Gammaproteobacteria, uncultured bacterium | 0.00025 |  | Geothermobacter | 0.000125 |
| Bergeyella | 0.00025 |  | Succinivibrionaceae, uncultured | 0.000125 |
| Acetobacteraceae, uncultured soil bacterium | 0.00025 |  | Planococcaceae, uncultured bacterium | 0.000125 |
| Kordiimonas | 0.00025 |  | Carnobacterium | 0.000125 |
| Proteus | 0.00025 |  | Marvinbryantia | 0.000125 |
| Alteromonadaceae, BD1-7 clade | 0.00025 |  | Candidate division JS1, uncultured | 0.000125 |
| SAR86 clade, uncultured marine bacterium | 0.00025 |  | Rhizobiales, uncultured soil bacterium | 9.96E-05 |


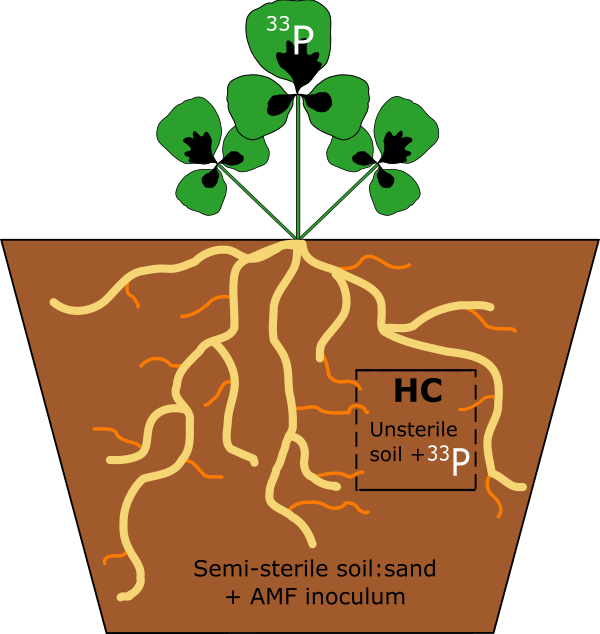


Figure S1. Diagram of model system used in all experiments: a hyphal compartment (HC) contained experimental unsterile soil labelled with a radioactive isotope of P, accessible only by AMF ERM, and not by roots. Roots and hyphae were able to grow in semi-sterile soil/sand mix, inoculated with AMF. Radioactive P was quantified in the shoots after harvest, in order to quantify the P uptake activity of ERM.

a)

b)

Figure S2. Relationship between shoot ^33^P content (log-transformed to conform to the assumption of normality) in *M. truncatula* plants grown in Expt. 2, and (a) HC soil pH or (b) HC water-extractable P concentration (log-transformed to conform to the assumption of normality) of 21 different unsterile soils (two replicates of each presented here). The significance and correlative strength of each relationship is denoted by the *P* and R^2^ values on each panel, respecitvely.


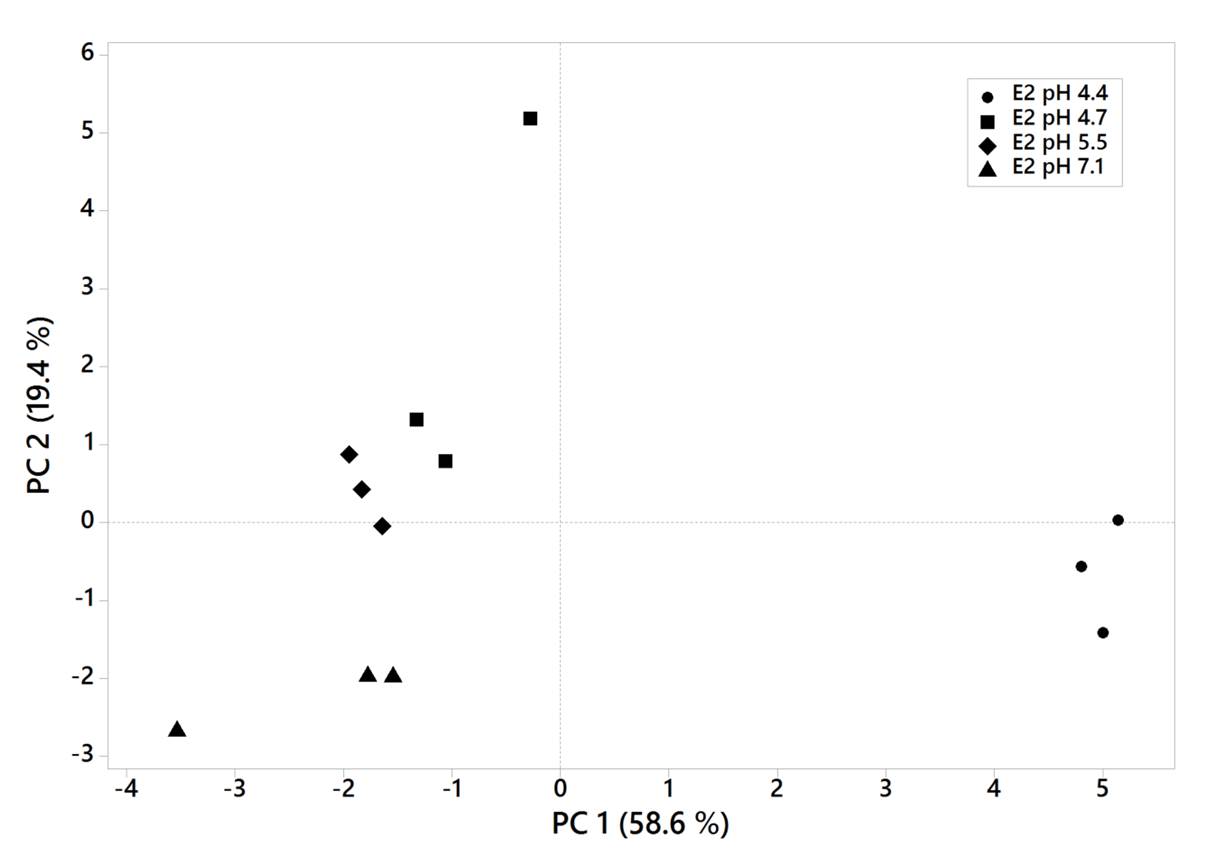


Figure S3. Principal Component Analysis score plot of microbial PLFAs from Møystad E2 exposed to liming treatments (Expt. 3).

(a)

ns

(b)

Figure S4. Diversity (Shannon H index) and richness (Chao-1) in samples from (a) Møystad E2 soil with pH 4.4 and limed to pH 7.1 and in (b) suppressive and conducive soils from Expt. 2.

(a)

(b)

Figure S5. Rarefaction curves for the number of observed OTUs with 97% similarity (a) in samples from Expt. 2 and (b) in samples from liming of Møystad E2 soil.


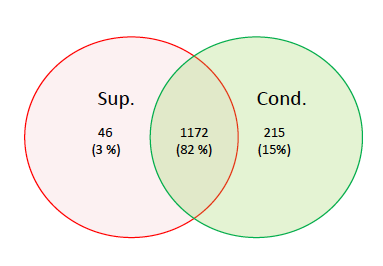


Figure S6. Venn diagram showing number of shared and unique genera in AMF-suppressive (red) and conducive (green) soils (Expt. 2).
